# Supplementary material for: Identification of exacerbation risk in patients with liver dysfunction using machine learning algorithms
Source: PLoS One. 2020 Oct 9;15(10):e0239266. doi: 10.1371/journal.pone.0239266 (PMC7546449; doi:10.1371/journal.pone.0239266)
Supplement: Attachment — Submitted filename: Response to Reviewers.docx [file pone.0239266.s002.docx]

Reviewers' comments:

Reviewer's Responses to Questions

**Comments to the Author**

1. Is the manuscript technically sound, and do the data support the conclusions?

The manuscript must describe a technically sound piece of scientific research with data that supports the conclusions. Experiments must have been conducted rigorously, with appropriate controls, replication, and sample sizes. The conclusions must be drawn appropriately based on the data presented.

Reviewer #1: No

Reviewer #2: Partly

2. Has the statistical analysis been performed appropriately and rigorously?

Reviewer #1: I Don't Know

Reviewer #2: I Don't Know

3. Have the authors made all data underlying the findings in their manuscript fully available?

The [PLOS Data policy](http://www.plosone.org/static/policies.action#sharing) requires authors to make all data underlying the findings described in their manuscript fully available without restriction, with rare exception (please refer to the Data Availability Statement in the manuscript PDF file). The data should be provided as part of the manuscript or its supporting information, or deposited to a public repository. For example, in addition to summary statistics, the data points behind means, medians and variance measures should be available. If there are restrictions on publicly sharing data—e.g. participant privacy or use of data from a third party—those must be specified.

Reviewer #1: No

Reviewer #2: Yes

4. Is the manuscript presented in an intelligible fashion and written in standard English?

PLOS ONE does not copyedit accepted manuscripts, so the language in submitted articles must be clear, correct, and unambiguous. Any typographical or grammatical errors should be corrected at revision, so please note any specific errors here.

Reviewer #1: Yes

Reviewer #2: Yes

5. Review Comments to the Author

Please use the space provided to explain your answers to the questions above. You may also include additional comments for the author, including concerns about dual publication, research ethics, or publication ethics. (Please upload your review as an attachment if it exceeds 20,000 characters)

Reviewer #1: Congratulations for the birlliant idea and excellent work for implication of machine learning to help caring patient with liver disease. My personal opinions are as following.


1. Generally speaking, the criteria for ICU admission were shock, respiratory failure or any major organ failure. What's the exact criteria for admission to intensive care unit, i.e. the criteria for high risk group. Were all the high risk group patients diagnosed of liver decompesation?
Reviewer #1-RESPONSE: Special thanks to you for your good comments. Inspired by your comments, we realized that there was a problem with study population selection. Thus, we resampled the research group. We labeled the patients those required the liver transplant due to the liver decompesation as the high risk group , while those did not require as low risk group. In this study, all the patients in both groups were admitted to hospital with impaired liver function.

2. In Table 1, consciousness was categorized as clear-minded and "Fuzzy". What's the definition of fuzzy conscousness? Does it mean irritable, agitated, hypersomnance, stupor or comatose? GCS?
Reviewer #1-RESPONSE: The state of consciousness includes clear awareness, fuzzy consciousness, drowsy, sleepy, shallow coma and deep coma . We have added the corresponding description in the second part of the paper.

3. In Table 1, Creatine(Cr) was included as a parameter, however, "patients with chronic kidney disease was excluded" according to the context. Is there specific definition, such as eGFR or MDRD for staging chronic kidney disease, and by which stage the patients were excluded?

Reviewer #1-RESPONSE: eGFR-MDRD is an indicator of chronic kidney disease in serum testing. The value of eGFR-MDRD can be calculated by weight, creatinine, and age. Serum creatinine is an important indicator of kidney function. In the new data collection, we do not exclude the patients with chronic kidney disease. We focused the patients those required the liver transplant due to the liver decompesation as the study population.

4. In Table 1, Prothrombin standardized ratio (INR) 2.6 1 was defined as low risk group. However, Child Pugh Score, a widely accepted scoring system for evaluation the liver status of cirrhotic patients, gives 2 ppoints for INR > 1.7.

Reviewer #1-RESPONSE: We are very sorry for our negligence that we made a mistake to select the patients those require ICU admission as the high risk group. We resampled the study population selection and select the patients those required the liver transplant due to the liver decompesation as the high risk group, while those did not require as low risk group. In this study, all the patients in both groups were admitted to hospital with impaired liver function. In the new collection, the INR in the low risk group was 1.39.

5. The goal of the current study was to forcast whether a patient would deteriorate after admission to the hospital. Defining "deteriorate", did the study focus only on liver function deterioration? As we know, a large proportion of patients with end staged liver disease sufferred from deteriorated condition due to events of renal failure, such as hepatorenal syndrome, or sepsis, such as SBP, or hypovolemic shock due to recurrent UGI bleeding (variceal bleeding), how did the study stratify different types of deterioration of liver function, is the etiology of deterioration considered?

Reviewer #1-RESPONSE:

As the reviewer described there were different types of deterioration of liver function. The complex deteriorated condition with end staged liver disease often exists due to the events of renal failure, such as hepatorenal syndrome, or sepsis, such as SBP, or hypovolemic shock due to recurrent UGI bleeding (variceal bleeding). Inspired by the reviewers’ insights, we resampled the study population by whether the patient has had a liver transplant with end staged liver disease as the high risk group, while those did not require as the low risk group. In fact, there are multiple severity ratings, and for practical operability, we identify classification as two categories (high risk and low risk)

6. Quoted from paragraph of Discussion: "Nevertheless,the assessment of liver failure based only MELD has some limitations, as the LD disease have a heterogeneous natural history and only a small 141 proportion of them actually cause clinical events." The current study did not stress some of the the well known features of VLD, such as HBV DNA level, HBsAg level or HBeAg/HBeAb for CHB flare. These established features of VLD were considered to play important role of liver function deterioration, and may be more predictive than some of the parameters as NEUT or TC.
Reviewer #1-RESPONSE: We checked the well known features of VLD in the raw data. We found that there were a lot missing values for the well known features such as HBsAg, HBeAg/HBeAb, HBV DNA level. The reason for missing values for the well known features may be that tests occurred before the current data, or for the other reasons. Considering the Reviewer’s profound opinion, we were concerned about the acute deterioration risk prediction in patients with the end staged liver disease caused by a variety of causes (viral liver, alcoholic liver, medicinal liver, pregnancy liver, etc.) using the common clinical indicators using machine learning methods.

7. Is it possible to share, at least part of the raw data and calculation of CART classifier? Then we can discuss the pitfalls of machine learning implication for liver disease.
Reviewer #1-RESPONSE: Thank you very much again for your profound comments, which have been of great help to our research work, and we will show you the entire desensitization data and procedures.

Thank you.

Reviewer #2: Peng et al. conducted a retrospective study to investigate the risk for patients with viral liver disease (VLD) who would deteriorate after admission to the hospital. A total of 308 liver dysfunction (LD) patients were identified, and among them, 283 VLD patients were included for analysis. The outcomes of VLD patients were dichotomously divided as high and low risk groups, and the high risk group was defined as those who required ICU care. They built the machine learning model by incorporating 9 key clinical indicators. Four different machine learning algorithms were utilized and their performance was compared to MELD scoring system. Finally, they claimed the performance of the proposed machine learning model is superior to the MELD model. Although machine learning is an interesting approach to help improve the risk stratification for VLD patients, some caveats of this study need to be carefully addressed in order to improve the quality.


1. The major issue is the definition of clinical outcomes (high and low risk groups) that were only based on the need of ICU care. It is uncertain whether the need of ICU care was due to liver-related causes or not. This needs to be clarified. Presumably, liver-related mortality or hepatic failure/the need for liver transplantation may be a more appropriate outcome for this analysis.

Reviewer #2-RESPONSE: Special thanks to you for your good comments. Inspired by your comments, we realized that there was a problem with study population selection. Thus, we resampled the research group. We labeled the patients those required the liver transplant due to the liver decompesation as the high risk group , while those did not require as low risk group. In this study, all the patients in both groups were admitted to hospital with impaired liver function.

2. Another issue is the choice of the 9 clinical indicators. There are quite a few number of clinical parameters associated with patients’ outcomes. What is the rationale for choosing only these 9 parameters. This should be described.

Reviewer #2-RESPONSE: Considering the Reviewer’s suggestion, we have added the rationale for choosing the clinical indicators. The principle of clinical indicators choice is to select the indicators with less missing values, and to select indicators with clinical significance among them.

3. As raised by the authors, no validation cohort is a concerning issue. Because this study has a small case number and 9 parameters for analysis, the chance of overfitting cannot be ignored.

Reviewer #2-RESPONSE: In order to overcome the chance of overfitting, we used 10 fold cross validation on the data set to make the model more general. In the future, we will collect more data for model improvement.

4. Based on the title of this paper, all viral hepatitis patients should be included. Why were only patients with hepatitis B and/or hepatitis E included? Hepatitis C should be also a common viral hepatitis. Was there any reason to exclude hepatitis C from this analysis?

Reviewer #2-RESPONSE: We are very sorry for our negligence that we did not state clearly our research objectives. In fact, we intended to predict deterioration risk of liver dysfunction using machine learning methods with the clinical indicators. The causes of liver decompesation include hepatitis B, hepatitis C, hepatitis E, drug-induced liver damage, autoimmune liver disease, liver bean-like nuclear degeneration, alcoholic liver disease, fatty liver disease during pregnancy, etc. We first collected the patients those required the liver transplant due to the liver decompesation as the high risk group , then collected those did not require as low risk group. In the new collection data, the majority of patients are hepatitis B virus patients, and a small number are Hepatitis C virus patients (only 6).

5. Whether patients have underlying liver disease is also an important factor for the severity of hepatitis episode. Was the hepatitis B in these patients acute or chronic? In addition, was advanced fibrosis or cirrhosis present before this episode of hepatitis?

Reviewer #2-RESPONSE: We resampled the research group. The high risk group consisted of the patients those required the liver transplant due to the liver disfunction. The causes of liver disfunction included acute or chronic liver disfunction or the other underlying liver disease.

6. What is the timing for collecting the 9 clinical indicators? Were they all collected upon admission? This may affect the results of this analysis.

Reviewer #2-RESPONSE: We collected the clinical indicators before treatment (liver transplant) after admission.

7. In this study, there were a total of 308 LD patients, whose clinical characteristics are shown in Table 1. However, it seemed that only 283 VLD patients were included for analysis. If this is the case, they should show the clinical characteristics of these 283 patients.

Reviewer #2-RESPONSE: We resampled the research group. The research group inclusion was whether a liver transplant had been performed. We focused on the deterioration risk prediction which caused a liver transplant using the clinical indicators. Thus, we are not concerned about the proportion of VLD patients in the research group.

8. In Table 2, ‘Na’ is missing.

 Reviewer #2-RESPONSE: We have added the description of ‘Na ‘.

6. PLOS authors have the option to publish the peer review history of their article ([what does this mean?](https://journals.plos.org/plosone/s/editorial-and-peer-review-process#loc-peer-review-history)). If published, this will include your full peer review and any attached files.


If you choose “no”, your identity will remain anonymous but your review may still be made public.


**Do you want your identity to be public for this peer review?** For information about this choice, including consent withdrawal, please see our [Privacy Policy](https://www.plos.org/privacy-policy).

Reviewer #1: Yes: Ta-Wei Liu

Reviewer #2: No


[NOTE: If reviewer comments were submitted as an attachment file, they will be attached to this email and accessible via the submission site. Please log into your account, locate the manuscript record, and check for the action link "View Attachments". If this link does not appear, there are no attachment files to be viewed.]


While revising your submission, please upload your figure files to the Preflight Analysis and Conversion Engine (PACE) digital diagnostic tool, <https://pacev2.apexcovantage.com/>. PACE helps ensure that figures meet PLOS requirements. To use PACE, you must first register as a user. Registration is free. Then, login and navigate to the UPLOAD tab, where you will find detailed instructions on how to use the tool. If you encounter any issues or have any questions when using PACE, please email us at [figures@plos.org](mailto:figures@plos.org). Please note that Supporting Information files do not need this step.
