# Supplementary material for: Identification of exacerbation risk in patients with liver dysfunction using machine learning algorithms
Source: PLoS One. 2020 Oct 9;15(10):e0239266. doi: 10.1371/journal.pone.0239266 (PMC7546449; doi:10.1371/journal.pone.0239266)
Supplement: Attachment — Submitted filename: Response to Reviewers.docx [file pone.0239266.s003.docx]

Reviewers' comments:

Reviewer's Responses to Questions

Comments to the Author

1. If the authors have adequately addressed your comments raised in a previous round of review and you feel that this manuscript is now acceptable for publication, you may indicate that here to bypass the “Comments to the Author” section, enter your conflict of interest statement in the “Confidential to Editor” section, and submit your "Accept" recommendation.

Reviewer #1: All comments have been addressed

Reviewer #2: All comments have been addressed

2. Is the manuscript technically sound, and do the data support the conclusions?

The manuscript must describe a technically sound piece of scientific research with data that supports the conclusions. Experiments must have been conducted rigorously, with appropriate controls, replication, and sample sizes. The conclusions must be drawn appropriately based on the data presented.

Reviewer #1: Partly

Reviewer #2: Yes

3. Has the statistical analysis been performed appropriately and rigorously?

Reviewer #1: I Don't Know

Reviewer #2: Yes

4. Have the authors made all data underlying the findings in their manuscript fully available?

The PLOS Data policy requires authors to make all data underlying the findings described in their manuscript fully available without restriction, with rare exception (please refer to the Data Availability Statement in the manuscript PDF file). The data should be provided as part of the manuscript or its supporting information, or deposited to a public repository. For example, in addition to summary statistics, the data points behind means, medians and variance measures should be available. If there are restrictions on publicly sharing data—e.g. participant privacy or use of data from a third party—those must be specified.

Reviewer #1: No

Reviewer #2: Yes

5. Is the manuscript presented in an intelligible fashion and written in standard English?

PLOS ONE does not copyedit accepted manuscripts, so the language in submitted articles must be clear, correct, and unambiguous. Any typographical or grammatical errors should be corrected at revision, so please note any specific errors here.

Reviewer #1: No

Reviewer #2: Yes

6. Review Comments to the Author

Please use the space provided to explain your answers to the questions above. You may also include additional comments for the author, including concerns about dual publication, research ethics, or publication ethics. (Please upload your review as an attachment if it exceeds 20,000 characters)

Reviewer #1: 1) Is the paper edited by native speaker of English? There were plenty of wording problems and grammar issues as:

- Grammar and misspelling : "Some liver diseasemay permanent liver 6

damage such as viral liver disease would lead to permanent liver failure"

"accuracyon"

- Missing words? "it is difficult to resolve the onset of the liver with some symptoms or indicators"

- Misspelling? "coagulation assase"

- In the paragraph of Feature Selection: "Conscience.Conscience.Different liver diseases..."

In terms of hepatoencephalopathy, usually "consciousness", instead of "conscience", is influenced, .

Reviewer #1-RESPONSE: Special thanks to you for your careful examination of our manuscripts. Inspired by your comments, we have checked the spelling and grammar of the whole paper carefully.

- Grammar and misspelling :

"Some liver diseasemay permanent liver 6 damage such as viral liver disease would lead to permanent liver failure" has been modified to “Some liver disease such as viral hepatitis would lead to permanent liver failure”.

"accuracyon" has been modified to "accuracy on"

- Missing words?

"it is difficult to resolve the onset of the liver with some symptoms or indicators" has been modified to “it is difficult to identify the onset of liver disease deterioration with some clinical symptoms or indicators”.

- Misspelling?

"coagulation assase" has been modified to “international normalized prothrombin ratio”.

- In the paragraph of Feature Selection: "Conscience.Conscience.Different liver diseases..."

In terms of hepatoencephalopathy, usually "consciousness", instead of "conscience", is influenced. “conscience” has been modified to "consciousness".

2) In the paragraph of Feature Selection: about "Calcium"

Quote: "maintaining the normal concentration of blood calcium plays an important role in the hormones are metformin, calcite and cholate calcinol."

As a matter of fact, metformin is an gluconeogenesis suppressor and insulin sensitizer, calcite is a carbonate mineral, and Calcinol is registered name of calcium supplement? It seems metformin, calcite and calcinol did not belong to the category of "hormone".

Reviewer #1-RESPONSE: We have revised the inappropriate statement “the hormones are metformin, calcite and cholate calcinol” to “Blood calcium levels are associated with many important functions of the human body. Parathyroid, calcitonin and cholecalcetol play an important role in maintaining normal blood calcium concentrations.”

3) Table 1, lacks the units of each parameter, such as AST, ALT, ALb, TPROT. What is "Ca", total calcium or ionized calcium. Glucosatosinase (AST) --> should be Aspartate transaminase? Glutamate transaminase (ALB) -> should be Albumin?

Reviewer #1-RESPONSE: We have the units of each parameter. "Ca" represents the total calcium and we have added the description in the feature selection section. Glucosatosinase (AST) has been modified to “Aspartate transaminase”. “Glutamate transaminase (ALB)” has been modified to “Albumin (ALB)”

4) In "Model Selection" (3) Generalized linear model (GLMs), f(y) = w^Tx + b;

The linear predictor was "w^Tx". The multiplier of x in the predictor was usually set to be constant, please indicate the role of the parameter "T" in the equation. By the way, the equation adopt "b" instead of theat as a natural threshold. The 15 indicators were continuos variables (except for consciousness), the GLMs which could be applied are: linear regression, ANCOVA, Poisson Regression or Multinomial response. Which GLM was choosed in this study? Actually the equation f(y) = w^Tx + b seems to describe the hyperplane of SVM.

Reviewer #1-RESPONSE: We have modified the description of Generalized linear model (GLMs) to “w is the weight vector, T denotes the operation of the vector transposition ,b represents the bias. f denotes the activation function which is determined by the training samples”. We checked and ran our procedures and found that binomial family was used as the contact function and the binomial family the links logit, probit, cauchit, (corresponding to logistic, normal and Cauchy CDFs respectively) log and cloglog (complementary log-log), thus, GLMs was applied as a logistic regression model. We are very sorry for our negligence that we did not state clearly the equation f(y) = w^Tx + b representing the common linear model.

5) Just curious, the authors used the CART to evaluate the 15 clinical indicators to idendify risk of liver failure, and 10 fold cross validation was used to prevent overfitting, why Random Forest was not applied in the first place?

Reviewer #1-RESPONSE: Random forest is generated by the ensemble of decision trees. It is widely used in the analysis and modeling of medical scenarios due to its rapidity, high accuracy, and robustness. However, real-world medical scenes usually require models with interpretability, random forest as a black box model can not meet the interpretability requirement. While the CART model can meet the interpretability requirement and achieve relatively high prediction accuracy by prevent overfitting. This is the reason why Random Forest was not applied in the first place.

6) In "Discussion", quote "There are some limits in our research. First, the present study used consciousness, 208 Cr, NEUT,ALT, TBIL, Na ,PA,INR and TC, etc, for the prediction of LF risk.". There were only 9 clinical indicators mentioned:　consciousness, Cr, NEUT,ALT, TBIL, Na ,PA,INR and TC. Were the other 6 indicators (AST, lymphocyte count, albumin, total protein, calcium and triglyceride) excluded and why?

Reviewer #1-RESPONSE: We are very sorry for our negligence that we did not state clearly the clinical indicators used in our research. In fact , we used the 15 clinical indicators in our research. We have modified the “First, the present study used consciousness, Cr, NEUT,ALT, TBIL, Na ,PA,INR and TC, etc, for the prediction of LF risk.” to “There are some limits in our research. First, the present study used conscience, AST, NEUT, LYMPH, CREAT, ALT, ALB, TPROT, TBILI, Na, Ca, PA, INR, CHOL and TRIG, for the prediction of LF risk”. Thank you very much again for your good comments, which have been of great help to our research work.

Reviewer #2: The authors have already satisfactorily addressed all of the concerns I raised. I have no more comments on this manuscript.

Thank you very much again for your good comments, which have been of great help to our research work.

7. PLOS authors have the option to publish the peer review history of their article (what does this mean?). If published, this will include your full peer review and any attached files.

If you choose “no”, your identity will remain anonymous but your review may still be made public.

Do you want your identity to be public for this peer review? For information about this choice, including consent withdrawal, please see our Privacy Policy.

Reviewer #1: No

Reviewer #2: No

[NOTE: If reviewer comments were submitted as an attachment file, they will be attached to this email and accessible via the submission site. Please log into your account, locate the manuscript record, and check for the action link "View Attachments". If this link does not appear, there are no attachment files.]

While revising your submission, please upload your figure files to the Preflight Analysis and Conversion Engine (PACE) digital diagnostic tool, https://pacev2.apexcovantage.com/. PACE helps ensure that figures meet PLOS requirements. To use PACE, you must first register as a user. Registration is free. Then, login and navigate to the UPLOAD tab, where you will find detailed instructions on how to use the tool. If you encounter any issues or have any questions when using PACE, please email PLOS at figures@plos.org. Please note that Supporting Information files do not need this step.
